# Efficacy and safety of mineralocorticoid receptor antagonists for patients with heart failure and diabetes mellitus: a systematic review and meta-analysis

**DOI:** 10.1186/s12872-016-0198-2

**Published:** 2016-01-29

**Authors:** Meng-Die Chen, Si-Si Dong, Ning-Yu Cai, Meng-Di Fan, Su-Ping Gu, Jin-Jue Zheng, Hai-Min Yin, Xin-He Zhou, Liang-Xue Wang, Chun-Ying Li, Chao Zheng

**Affiliations:** Diabetes Center and Department of Endocrinology, The Second Affiliated Hospital of Wenzhou Medical University, No. 109 West Xueyuan Road, 325027 Wenzhou, Zhejiang China; Department of Orthopedics, The Second Affiliated Hospital of Wenzhou Medical University, 325027 Wenzhou, Zhejiang China

**Keywords:** Mineralocorticoid receptor antagonists, Heart failure, Diabetes mellitus

## Abstract

**Background:**

The aim of this study was to systematically assess the efficacy and safety of mineralocorticoid receptor antagonists (MRAs) for patients with heart failure (HF) and diabetes mellitus (DM).

**Methods:**

We conducted a comprehensive search for controlled studies that evaluated the efficacy and safety of MRAs in patients with DM and HF. Medline, Embase and Cochrane databases were searched. Two reviewers independently identified citations, extracted data and evaluated quality. Risk estimations were abstracted and pooled where appropriate.

**Results:**

Four observational studies were included. MRAs use was associated with reduced mortality compared with controls (RR = 0.78; 95 % CI: 0.69–0.88; I ^2^ = 0 %; *P* < 0.001). Increased risk of developing hyperkalaemia was observed in those patients taking MRAs (RR = 1.74; 95 % CI: 1.27–2.38; I ^2^ = 0 %; *P* = 0.0005).

**Conclusions:**

The current cumulative evidence suggests that MRAs can improve clinical outcomes but increase the risk of hyperkalaemia in patients with DM and HF.

**Trial registration:**

PROSPERO CRD42015025690.

**Electronic supplementary material:**

The online version of this article (doi:10.1186/s12872-016-0198-2) contains supplementary material, which is available to authorized users.

## Background

Diabetes mellitus (DM) and heart failure (HF) commonly coexist. About 40 % of hospitalized HF patients have DM, and these figures are expected to grow with the general aging of the population [1]. Results from more than 100,000 patients in the Acute Decompensated Heart Failure National Registry suggested that 44 % of HF patients had DM [2]. Results from a health maintenance organization show that about 12 of 10,000 patients with DM had HF at baseline, and 3.3 % of the rest developed HF during each year of follow-up [3]. There is now a large number of epidemiological and clinical data supporting the strong association between HF and DM [4]. Patients with HF can have insulin resistance, which increases their risk of developing type 2 DM [5]. It was shown in an Italian observational study that 28 % of elderly patients with HF developed new-onset type 2 DM in 3 years and HF is an independent risk factor for type 2 DM (OR 3.3; 95 % CI 2.6–4.0) [6]. Patients with HF are not only at increased risk of developing DM but patients with DM also have a greater probability of developing HF [7]. In patients with DM, every unit increase in glycosylated hemoglobin (HbA1c) is associated with a 10 % to 15 % increased risk of developing HF [8]. Recent literature suggested that co-existence of DM and HF can lead to increased morbidity and mortality [9]. Hospitalized HF patients with DM have an even worse prognosis with increased rates of cardiovascular (CV) mortality and post-discharge HF hospitalization [10]. Recently, a subgroup analysis of the results indicated that during standard treatment, side effects were most likely to appear in hospitalized HF patients with DM compared to those without DM [11]. Thus, treating coincident HF and DM is still a challenge.

Mineralocorticoid receptor antagonists (MRAs) are powerful treatment agents for patients with cardiovascular disease [12]. Morbidity and mortality benefits from treatment with MRAs have been demonstrated in HF patient and MRAs have become part of standard medical therapy for HF [13, 14]. Similar to HF patients without DM, treatment with MRAs is associated with improved outcomes in patients with DM [15]. However, associated adverse events including hyperkalaemia, gynecomastia, menstrual irregularities, and acute kidney injury can not be ignored [16]. The effects of MRAs on glycaemic control are still uncertain. The results of some studies have demonstrated that spironolactone significantly elevated HbA1c levels or worsened glycaemic control [17, 18], while one study has shown that spironolactone may have a beneficial effect on serum insulin and HOMA-IR in patients with non-alcoholic fatty liver disease [19]. A few studies support the view that MRAs, whether spironolactone or eplerenone, did not have a significant effect on glucose levels [20–22]. Furthermore, results of a small direct comparative trial have shown that spironolactone increased HbA1c in patients with DM and HF, but eplerenone did not [23].

Aldosterone is a mineralocorticoid hormone that activates the apical epithelial sodium channel and the basolateral Na^+^ /K^+^ ATPase pump, and controls sodium excretion at the level of the distal tubules to exert an action on sodium homeostasis [24]. However aldosterone can have harmful effects on the cardiovascular system [25]. By blocking the mineralocorticoid receptor in the distal tubule of the kidney, MRAs prevent the activation of sodium channels and lead to diuresis with reduced excretion of potassium [26]. MRAs can prevent vascular inflammation, myocardial fibrosis and ventricular remodelling, and improve endothelial function [14]. HF and DM are characterized by a high level of oxidative stress and it has been reported that MRAs can reduce oxidative stress [18].

A better understanding of the efficacy and safety of MRAs in patients with HF and DM is needed. To date, no meta-analysis has been conducted concerning the relationship between treatment with MRAs and outcomes in those patients. Therefore, it is worth undertaking a systematic review to assess the efficacy and safety of treatment with MRAs in patients with concomitant HF and DM.

## Methods

This systematic review was conducted according to the Preferred Reporting Items for Systematic Reviews and Meta-Analyses (Additional file 1) guidelines [27].

### Eligibility criteria

#### Types of studies

A study was included if it was either a randomised or non-randomised controlled trial, a prospective or retrospective cohort study, or a case–control study that evaluated the efficacy and safety of MRAs in patients with HF and DM.

#### Types of participants

Patients of any age, gender or race with HF and DM were included.

#### Types of interventions

Patients in the treatment groups were given MRAs while the control groups were given placebo or had no intervention. The studies were included regardless of the follow-up duration and dosage of treatment.

#### Types of outcome measures

The primary outcome measures were all-cause mortality and hyperkalaemia. The secondary outcome measures were CV mortality or HF hospitalization, death from CV causes and change in estimated glomerular filtration rate (eGFR).

### Literature search

A computerized literature search was conducted using Medline (1966–2015), Embase (1980–2015) and the Cochrane Central Register of Controlled Trials (1991–2015) from their inception to July 2015. The search strategy in Medline is shown in Additional file 2. In addition, we manually searched reference lists of all included studies and relevant review articles. Furthermore, we contacted experts and authors of included studies to retrieve potentially relevant published or unreported material. The retrieval was not restricted by language or quality of study.

### Study selection and data collection

Two reviewers (G.S.P. and Y.H.M.) independently screened the titles and abstracts to select potential references according to a data extraction form established above, including patients, methods, interventions and outcomes. For eligible studies, two reviewers (G.S.P. and Y.H.M.) extracted the data independently. Disagreements were settled through discussion or consultation with a third author (F.M.D.).

### Quality assessment

The quality of the included studies was evaluated using the nine-star Newcastle Ottawa scale (NOS) [28] by two investigators. Each study was evaluated based on eight items, and the scale ranged from zero to nine stars, the more stars the higher the methodological quality.

### Data synthesis and analysis

Data were analyzed using Review Manager (version 5.2). A fixed-effects model or random-effects model was used across the studies; relative risk (RR) and 95 % confidence intervals were calculated for dichotomous outcomes. A standard chi-square test and I^2^ statistic were calculated to evaluate the heterogeneity between trial results. All P values were two sided and P values of less than 0.05 for any test were considered to be statistically significant.

## Results

### Study selection

**Fig. 1:**
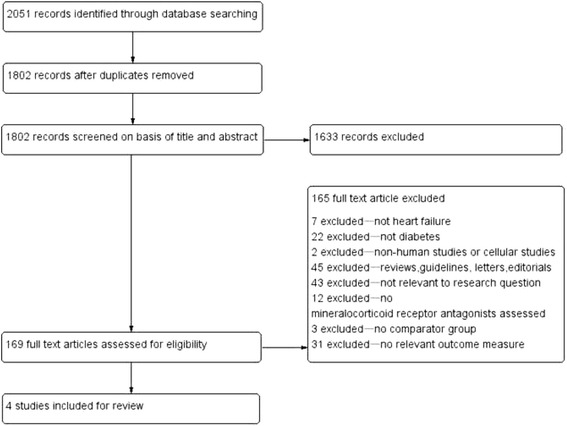
Flow diagram of study selection. Identified were 2051 potentially relevant articles; 1802 were excluded through screening of titles and abstracts, and 165 articles were excluded for not meeting our inclusion criteria. Finally, 4 studies were included

There were 2051 potentially relevant articles retrieved of which 1802 were excluded because they were case reports, animal or basic research studies, or did not investigate any of outcomes of interest. After screening the titles and abstracts, we excluded 165 articles of the remaining 169 articles, for not meeting our inclusion criteria. No more eligible studies were found after manually searching reference lists. Ultimately a total of 4 studies that included 4742 participants were included in this systematic review. The screening process is summarized in a flow diagram (Fig. 1).

### Study characteristics

No randomized controlled trials were identified. Three [29–31] were post hoc subgroup analyses from randomized trials and one [32] was a prospective cohort study. A total of 4742 participants were included in the 4 studies, of whom 2188 were in treatment groups and 2554 were in control groups. In two studies [29, 30], the control medicine was placebo (*n* = 1134); and the remaining two studies [31, 32] comparison groups without MRAs (*n* = 1420) were used.

Three studies [29–31] contained additional HF-specific information (e.g., New York Heart Association Functional Class or LVEF). These also included demographics, co-morbidities, other related drug therapies and additional laboratory determination data. One study [32] was an administrative analysis with no additional laboratory or clinical information. Detailed characteristics of included studies are listed in Table 1.

**Table 1:** Characteristics of the included studies

Study	Subjects (T/C)	Age (T/C)	Male (T/C)	Ejection fraction, (%) (T/ C)	Interventions (T/C)	Follow-up period	Outcomes	Crude events (T/C)
O’Keefe (2007) [29]	749/734	66 ± 10/66 ± 10	63/64	32 ± 6 /32 ± 6	Eplerenone/Placebo	Mean 16 months	①all-cause mortality	①153/175
							②Hyperkalaemia(>5.5 mmol/l)	②42/22
							③Death from CV causes	③131/152
Eschalier (2013) [30]	459/400	68.1 ± 7.4/N.r.	356/N.r.	26.44 ± 4.7/N.r.	Eplerenone/Placebo	N.r	①Hyperkalaemia(>5.5 mmol/l)	①63/33
							②CV mortality+HF hospitalization	②99/141
							③Change in eGFR from baseline to final visit	③- 4.94(17.4)/-2.93(18.9)
Vadugana than (2014) [31]	444/306	65.1 ± 10.1/67.9 ± 10.7	320/242	26.7 ± 7.8/28.6 ± 8.8	MRAs/no MRAs	Mean 9.9 months	①all-cause mortality;	①101/98
							②CV mortality+HF hospitalization	②182/147
							③Death from CV causes	③78/66
Khosraviani (2014) [32]	536/1114	N.r.	N.r.	N.r.	Spironolactone/no spironolactone	2 years	all-cause mortality	79/223

Notes: *T* Trial Group, *C* Control Group, *N.r*. not report, *MRAs* mineralocorticoid receptor antagonists, *CV* cardiovascular, *HF* heart failure, *CI* confidence interval, *eGFR* estimated glomerular filtration rate

### Risk of bias in included studies

The methodological quality of the included studies is summarized in Table 2. The NOS results showed an average overall score of 7.25 (range 5–9).

**Table 2:** Newcastle-Ottawa Scale (NOS) assessment of the quality of the studies

	Selection				Comparability			Outcome		Score
Study	1	2	3	4	5a	5b	6	7	8	
O’Keefe, et al (2008) [29]	★	★	★	★	★	★	★	☆	★	9
Eschalier, et al (2013) [30]	★	★	★	★	☆	☆	★	☆	★	6
Vaduganathan, et al (2014) [31]	★	★	★	★	★	★	★	☆	★	9
Khosraviani, et al (2014) [32]	★	★	★	★	☆	☆	★	☆	☆	5

Notes: *1* indicates exposed cohort truly representative, *2* the non exposed cohort drawn from the same community, *3* ascertainment of exposure by secure record or structured interview, *4* outcome of interest was not present at start of study, *5A* cohorts comparable on basis of sex and age, *5B* cohorts comparable on other factor(s), *6* quality of outcome assessment, *7* follow-up long enough for outcomes to occur, and *8* complete follow up, ★ yes, ☆ no

### Effectiveness

#### All-cause mortality

**Fig. 2:**
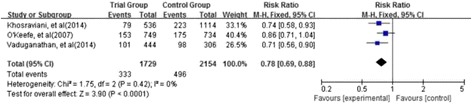
Forest plot of the comparison of treatment with MRAs versus without MRAs on all-cause mortality. Three of included studies evaluated the effect of MRAs on all-cause mortality. The mortality was 19 % in MRA groups compared with 23 % in control groups. The studies suggested that MRAs-based regimens reduced the risk of all-cause mortality in comparison to regimens without MRAs

Three of included studies [29, 31, 32] evaluated the effect of MRAs on all-cause mortality; 2-year, 16-month and 9.9-month mortality were evaluated respectively in these studies. Overall, the mortality was 19 % in the MRA treatment groups compared with 23 % in control groups (RR = 0.78, 95 % CI: 0.69–0.88, I ^2^ = 0 %, *P* < 0.001; Fig. 2). All studies suggested MRA-based regimens reduce the risk of all-cause mortality in comparison to regimens without MRAs.

In a study by O’Keefe et al. [29], which was a post hoc analysis from the EPHESUS trial, a reduction in all-cause mortality was observed in the eplerenone group that did not reach statistical significance. Khosraviani et al. [32] observed that spironolactone significantly reduced mortality compared to the control group without spironolactone (14.8 vs. 20.0 %, RR 0.74 [95 % CI 0.58–0.93]). Vaduganathan et al. [31] observed that MRA administration was associated with a 31 % reduction in all-cause mortality (RR 0.71 [95 % CI 0.56–0.90]) in unadjusted analyses, but the result turned to be negative after adjusting for baseline risk factors (adjusted HR 0.93; 95 % CI 0.75 to 1.15).

#### CV mortality or HF hospitalization

**Fig. 3:**
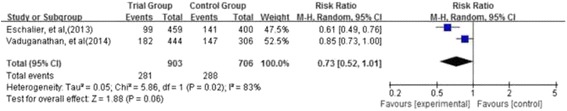
Forest plot of comparison of treatment with MRAs versus without MRAs on cardiovascular mortality or heart failure hospitalization. Two studies evaluated the effect of MRAs on CV mortality or HF hospitalization. Events occurred in 281 of the 903 participants treated with MRAs (31.1 %) compared with 288 of 706 (40.8 %) in the control group. Because significant heterogeneities were detected, we used a random-effect model to synthesize the data on the basis of the large population. These results did not reach statistical significance

Two studies [30, 31] evaluated the effects of MRAs on CV mortality or HF hospitalization. Events occurred in 281 of the 903 participants treated with MRAs (31.1 %) compared with 288 of 706 (40.8 %) in the control group. Because significant heterogeneities were detected, we used a random-effect model to synthesize the data (RR = 0.73; 95 % CI: 0.52–1.01; I ^2^ = 83 %; *P* < 0.06; Fig. 3). However, these results did not reach statistical significance.

Eschalier et al. [30] observed that the HR as the primary outcome in the eplerenone group compared with the placebo group was 0.61 (95 % CI: 0.49 to 0.76). Vaduganathan et al. [31] observed that MRA treatment was associated with a 19 % reduction in the end point (RR 0.85; 95 % CI 0.73 to 1.00) in unadjusted analyses, but the results became negative after adjusting for baseline risk factors (adjusted HR 0.94; 95 % CI 0.80 to 1.10).

#### Death from CV causes

**Fig. 4:**
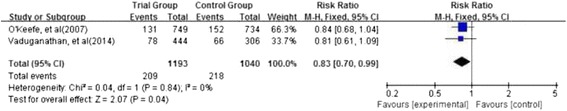
Forest plot of comparison of treatment with MRAs versus without MRAs on death from cardiovascular causes. Two studies evaluated the effect of MRAs on death from cardiovascular causes. Treatment with MRA-based regimens was associated with a statistically significant reduction in CV mortality compared with other treatments. Individually, in no study were observed statistically significant reductions in CV-cause mortality

Two studies [29, 31] evaluated the effect of MRAs on death from cardiovascular causes. Treatment was associated with a statistically significant reduction in CV mortality compared with control group (17.5 % versus 20.9 %; RR = 0.83; 95 % CI: 0.70–0.99; I^2^ = 0 %; *P* = 0.04; Fig. 4). Individually, no study observed statistically significant reductions in CV mortality.

### Safety

#### Hyperkalaemia

**Fig. 5:**
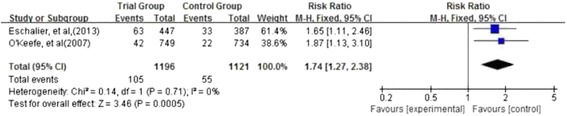
Forest plot of comparison of treatment with MRAs versus without MRAs on hyperkalaemia. Two studies evaluated the risk of developing hyperkalaemia caused by MRAs in patients with DM and HF. The occurrence of hyperkalaemia in the MRA group was higher than in the comparison group

Two studies [29, 30] evaluated the risk of developing hyperkalaemia caused by MRAs in patients with HF and DM. The occurrence of hyperkalaemia in the MRA group was higher than in the comparison group (8.7 % versus 4.9 %; RR = 1.74; 95 % CI: 1.27–2.38; I^2^ = 0 %; *P* = 0.0005; Fig. 5).

Eschalier et al. [30] observed there was an increase in the incidence of potassium levels of >5.5 mmol/l with eplerenone (14.1 %) in patients with DM and HF compared with placebo (8.5 %), *P* = 0.01. O’Keefe et al. [29] also observed that hyperkalaemia occurred more frequently with eplerenone treatment than with placebo (5.6 vs. 3.0 %, *P* = 0.015).

#### Change in eGFR

The change in eGFR from baseline to after the treatment period was reported in one study [30]. No significant difference was observed in the two groups (*P* > 0.05).

## Discussion

Four studies with 4742 individuals were identified. The main findings of the present study were that MRA treatment was associated with improved clinical outcomes compared with those regimens without MRAs in patients with DM and HF. From a safety perspective, the most serious adverse effect of MRAs (spironolactone and eplerenone) is the development of hyperkalaemia. However, as adverse effects were mentioned in only 50 % of the studies, there was not sufficient evidence to draw conclusions on the issue of safety.

There are a number of limitations in this study. First, only four studies were available; the included studies were all observational and none of them were randomized controlled trials. There were three post-hoc sub-analyses of randomized controlled trials concerning MRAs in heart failure and just one prospective cohort study. Two students scored the quality of each of the articles, and quality scores for each study are shown in Table 2. However, the quality of individual research included in our analysis was not necessarily high. Eschalier et al. [30] did not provide the detailed information about characteristics of the subject investigated. Khosraviani et al. [32] failed to offer an elaborated description of the study design. Thus, the level of evidence for this meta-analysis does not seem to be high.

Secondly, any systematic review may suffer from publication or selection bias. Publication bias can be assessed graphically with a funnel plot, which could not be assessed owing to having just four available studies. Thirdly, methodology defects have been found in some of these studies, including failure to collect data prospectively and inadequate baseline comparisons. Some baseline characteristics were different among the studies. For example, studies used different MRAs (spironolactone, eplerenone), different controls (placebo control or blank control), different dosages of the active and control medicines, and different follow-up times and background therapies. Lastly, significant heterogeneities were detected when the effects of MRAs on CV mortality or HF hospitalization were evaluated. The heterogeneity among the studies might be affected by various factors, such as study designs, study quality and patient characteristics. Therefore, a random-effect model was used to synthesize the data. But because only two studies reported these events, we could not perform meta-regression meta-analysis to examine the source of the heterogeneity.

Due to the limited quantity and quality of the included studies, it is premature to draw conclusion about the efficacy of MRAs in patients with HF and DM. However, it should be noted that the absence of sufficient scientific evidence does not mean that the treatment is ineffective. The safety of MRA therapy for those patients remains to be further determined. The possible electrolyte trouble could have a multifactorial origin. We observed that when using MRAs, many patients also used others drugs, including ACE inhibitors, angiotensin receptor blockers (ARBs), beta-blockers, and aspirin, but medical therapy was not different at baseline between the two groups. When the treatment with MRAs was undertaken, ACE inhibitors or ARBs were generally not discontinued, but patients underwent a modulation of their dosages or their dose was left unchanged. This suggests that ACE inhibitors or ARBs could be associated with the increased risk of hyperkalaemia; MRAs alone might not have caused this adverse effect. At the stage of the addition of MRA to therapy, a gradual up-titration of the MRA dose or a concomitant thoughtful reduction of the dose of ACE inhibitor or ARB could prevent the occurrence of hyperkalaemia, except in cases of severe impairment of kidney function. In addition, DM is an independent risk factor for the development of hyperkalaemia [33].

Several implications for research arise from this review. First, more randomized controlled trials of higher quality, with larger sample size and adequate follow up are needed to further evaluate the effect of MRAs. Second, the only extensively researched MRAs to date are spironolactone and eplerenone, further research on other MRAs such as canrenone is encouraged in these disease areas. Third, we should closely observe the incidence of adverse events, such as electrolyte abnormalities, gynecomastia, menstrual irregularities, acute kidney injury and the effects on serum glucose levels. Fourth, further study can focus on developing novel MRAs that have similar outcomes as spironolactone but lower rates of hyperkalaemia such as finerenone [34, 35]. Fifth, further study is needed with a focus on evaluating the outcomes of efficacy and safety in patients with heart failure associated with chronic renal failure, especially in patients with diabetic nephropathy, which are better represented in the literature regarding MRA efficacy and safety.

## Conclusions

The available evidence suggests that MRAs can reduce mortality risk in patients with HF and DM. However, the development of hyperkalaemia was reported in some of the reviewed studies. There is still a lack of convincing evidence for further evaluation of this treatment. More randomized controlled trials of higher quality, with a larger sample size, and long-term follow-up are needed in the future.

## Additional files

Additional file 1:
**PRISMA 2009 Checklist.** (DOC 54 kb) Additional file 2:
**Search strategy for Medline.** (DOCX 11 kb)

## Abbreviations

MRAs: Mineralocorticoid receptor antagonists
DM: Diabetes mellitus
HF: Heart failure
EF: Ejection fraction
CV: Cardiovascular
HbA1c: Glycosylated hemoglobin
eGFR: Estimated glomerular filtration rate
RR: Relative risk
