# Supplementary material for: Efficacy and safety of mineralocorticoid receptor antagonists for patients with heart failure and diabetes mellitus: a systematic review and meta-analysis
Source: BMC Cardiovasc Disord. 2016 Jan 29;16:28. doi: 10.1186/s12872-016-0198-2 (PMC4731899; doi:10.1186/s12872-016-0198-2)
Supplement: Additional file 2: — Search strategy for Medline. (DOCX 11 kb) [file 12872_2016_198_MOESM2_ESM.docx]

**Additional file 2: Search strategy for Medline**

#1 "Mineralocorticoid Receptor Antagonists"[Mesh]

#2 "mineralocorticoid receptor antagonists" OR "mineralocorticoid antagonists" OR "aldosterone antagonists" OR "aldosterone receptor antagonists" OR spironolactone

#3 "Heart Failure"[Mesh]

#4 "heart failure" OR "cardiac failure" OR "heart decompensation" OR "myocardial failure"

#5 "Diabetes Mellitus"[Mesh]

#6 diabet*

#7 #1 OR #2

#8 #3 OR #4

#9 #5 OR #6

#10 #7 AND #8 AND #9
